# Supplementary material for: iCollections methodology: workflow, results and lessons learned
Source: Biodivers Data J. 2017 Sep 25;(5):e19893. doi: 10.3897/BDJ.5.e19893 (PMC5665009; doi:10.3897/BDJ.5.e19893)
Supplement: Supplementary material 1 — NHM Imaging Standards [file bdj-05-e19893-s001.pdf]

Imaging standards

Introduction. Purpose of imaging

This document establishes principles of capture, documenting, and assessing quality of images taken at the NHM in the course of digitisation. This is a working document and it will be amended and corrected to accommodate changes in technology and digitisation practices.

Objects

2D artefacts – still image materials (Metamorfoze and FAGDI guidelines apply)

Image quality standards for digitisation of 2D cultural materials have been extensively discussed and accepted both in Europe [1] and North America [2]. Image quality criteria used in the Global Plant Initiative are roughly analogous to the Metamorfoze Extra Light standard and should be followed in herbarium sheet digitisation.

Natural history objects and 3D artefacts

The document deals primarily with 3D natural history objects and artefacts from the NHM's collection. Unlike cultural materials, there are no current standards developed for imaging natural history objects. Standards should be established following current recommendations on "per project" basis based on the intended use of images, necessary equipment and techniques, and limiting factors for the project. For each particular project, the minimum necessary quality criteria must be established and followed. The highest level for each quality criteria, which does not compromise objectives of the project, should be selected.

Use of images

The use of images may include but not limited to:

- Mass digitisation projects
- Label transcription
- Digital loans
- Taxonomic research
- Quantitative analysis
- Web publication
- Commerce

Equipment used

Examples of equipment used are:

- Conventional photographic cameras
- Scanners
- Scientific cameras and instruments, such as light microscopes
- Specialised imaging (non-visible spectrum)

---

[1] Hans van Dormolen, *Metamorfoze Preservation Imaging Guidelines. Image Quality, Version 1.0*.

[2] The FADGI Still Image Working Group, *Guidelines: Technical Guidelines for Digitizing Cultural Heritage Materials*.

- 3D visualisation

The name of a particular piece of equipment must be saved in the metadata (see Metadata). Technical descriptions, specifications, calibration profiles, and protocols must openly accessible.

Constraints

The most important limiting factors must be identified for each particular project.

Speed

For mass digitisation projects speed may be prioritised over image quality.

Aesthetic perception

For production: printing, retail, competitions. The speed of imaging and post-processing may be compromised in order to improve perceptual image quality.

Research integrity

For quantitative or experimental research projects. The reproducibility of results is paramount, thus both speed of acquisition and metadata collection will be lower in these projects. Raw data must always be saved with interpreted data. Quality must be evaluated through ground truth data (using targets, standard calibrations, etc.)

Storage destination

The nature of the objects and images identifies the final storage destination, which, in turn, affects image acquisition and metadata collection workflows.

KE EMu

All images of Museum specimens (and supplementary images) must be stored in KE EMu and associated with the corresponding specimen record. Images of specimens from other sources may be ingested into EMu at the curator's discretion. Images can be ingested manually or through a batch process; in the latter case, proper naming conventions and metadata schema must be followed.

MAMS

Images not related to museum specimens or intended for commercial use must be stored in the Media Asset Management system (MAMS). Ingest procedures are to be clarified when the MAMS project is completed.

Unified project folder

Research project images that cannot be stored in EMu (for example multidimensional proprietary formats such as CT-scans, or XRF, EDX analyses data, etc.) must be stored on the network hierarchical storage area ("unified"). Each experiment must be accompanied by complete set of technical metadata to ensure reproducibility. Folder structure:

/unified/instrument/date/person/project

/unifies/department/person/project

Quality levels table

Table 1. Image capture quality criteria

Image capture quality criteria		Quality levels: For each level speed (S), perceptual quality (P) and research (R) integrity is assessed: -: not acceptable, *: acceptable under certain circumstances, **: good; ***: excellent; n: irrelevant – 1	2	3
**Pre-acquisition**	Device-level calibration	omitted S***/P***/R*	Profiling using Colour Checker S**/P**/R**	Profiling using Universal Test Target or Device Target S*/P***/R***
	Monitor calibration	Using tools supplied with operating system S***/P*/R**	Initial calibration using simple colorimeter S**/P**/R***	Regular calibration and profile update using professional colorimeter S*/P***/R**
**Composition**	Framing	no special framing S***/P*/R**	specimen/structure occupies most of the frame S**/P***/R***	
	Magnification	single magnification, no framing S**/P*/R*	several constant magnifications, periodical framing S*/P*/R**	continuous magnification, framing for each image S*/P***/R***s
	Label imaging	not required/not implemented S***/Pn /R*	Labels images as a separate process S**/Pn/R**	Labels images simultaneously or parallel to specimens S*/Pn/R**
	Focusing	autofocus S*/P*/R**	focusing strategy S**/Pn/R**	manual focus S***/P***/R**
**Acquisition**	Illumination, evenness	visually assessed S**/P**/R*	corrected in post-processing S*/P***/R-	corrected through calibration profile S***/P***/R***
	Light source	visually assessed S**/P*/R*	Known colour temperature and spectral profile S**/P**/R**	D50 or D65-compatible bulbs, high-reflectance diffusion, calibration profile S***/P***/R***
	Scale Bar	omitted or estimated through software S**/Pn/R*	physical scale in every image S*/Pn/R*	virtual scale for precisely calibrated instruments S***/Pn/R***
	Camera ISO	automatic Sn/P**/R*	native ISO Sn/P*/R**	native ISO and all changes to gain documented Sn/P*/R***
	White balance	automatic white balance S*/P**/R*	manual white balance set on white paper or colour temperature set approximately S**/P**/R**	manual white balance set on spectrally neutral grey or to exact colour temperature using photometer S**/P***/R***

	Tonal range/exposure	automatic exposure S*/P**/R*	Histogram S**/P**/R**	Calibrated using device-level target, object-level target in every image S*/P**/R***
	Colour accuracy	automatic colour profile S**/P**/R**	Colour checker is used to create a universal profile S**/P**/R***	Calibrated using standard targets, colour checker in every image S*/P*/R***
	Background correction	ignored S**/P*/R*	corrected in post-processing S*/P**/R-	corrected quantitatively and documented S*/P***/R***
	Noise	ignored S**/P*/R*	corrected in post-processing S*/P**/R-	corrected quantitatively and documented S*/P***/R***
	Chromatic aberrations	ignored S**/P*/R*	corrected in post-processing S*/P**/R-	corrected quantitatively and documented S*/P***/R***
	Colour space	sRGB S**/P**/R**	sRGB or AdobeRGB S**/P***/R**	ProPhotoRGB (for 16 bit images) S*/P***/R***
	Bit depth	8-bit colour, 8-bit greyscale S***/P**/R**	8-bit colour, 16-bit greyscale S**/P**/R***	16-bit colour, 16-bit greyscale S*/P***/R***
	File formats	JPEG, 100% quality S***/P**/R*	TIFF, LZW or ZIP compressed S**/P***/R**	RAW S*/P***/R***
	RAW format	not acquired S***/P*/R*	discarded after saving in lossless format S**/P***/R*	preserved together with interpreted files S*/P**/R***
	Sampling rate (scanners)	See notes		
	Depth of field			
	Aperture			
	Resolution			
	Pixel array			

Table 2. Image post-processing

**Post-Processing**		**File formats**		
		**JPEG**	**TIFF**	**RAW**
**Image manipulation**	Master files	Kept safe and unchanged, for all manipulations use a copy of data S**/P***/R***		
	Lens distortion	Acceptable on a copy of original file S*/P***/R**		
	Global adjustments of brightness and contrast, levels, and gamma	Acceptable on a copy of original file as long as no information is lost (intensity histogram is not overstretched) S**/P***/R**		Acceptable on original file as long as editing is

			non-destructive S**/P**/R**
	Cropping	Acceptable as long as does not affect software-estimated scales S*/P***/R**	
	Local adjustments (dodging or burning)	Not acceptable S*/P***/R-	
	Local edits (clone, eraser)	Not acceptable unless for a strong reason; must be documented S*/P***/R-	
	Scaling (changing of size of image in pixels)	Not acceptable S**/P***/R-	
	Artificial colouring	Acceptable for greyscale images; must be documented S*/P***/R*	
**Reconstruction**	Extended Depth of Field (z-stacking)	see notes	
	Tiling (panorama)		
	Volumetric reconstruction		

Table 3. Metadata requirements

**Metadata**		**Quality levels**		
		**1**	**2**	**3**
**Audubon Core schema**	Identifier	file name or folder structure S**/P**/R*	EXIF/IPTC/XMP (see notes) S**/P***/R***	AudubonCore compatible description in CMS
	Type	Text document stored with files	EXIF/IPTC/XMP	AudubonCore compatible description in CMS
	Title	Text document stored with files	EXIF/IPTC/XMP	AudubonCore compatible description in CMS
	Metadata Language	Text document stored with files	EXIF/IPTC/XMP	AudubonCore compatible description in CMS
	Copyright Owner	Text document stored with files	EXIF/IPTC/XMP	AudubonCore compatible description in CMS
	Copyright Statement	Text document stored with files	EXIF/IPTC/XMP	AudubonCore compatible description in CMS
	Non-mandatory AC fields	Text document stored with files	EXIF/IPTC/XMP	AudubonCore compatible description in CMS
**Instrument/method related**	Instrument name	file name or folder structure	EXIF/IPTC/XMP	AudubonCore compatible description in CMS
	Description of protocols	Text document stored with files	EXIF/IPTC/XMP	AudubonCore compatible description in CMS

**Project related**	Team/department	file name or folder structure	EXIF/IPTC/XMP	AudubonCore compatible description in CMS
	Person	file name or folder structure	EXIF/IPTC/XMP	AudubonCore compatible description in CMS
	Project name	file name or folder structure	EXIF/IPTC/XMP	AudubonCore compatible description in CMS
	Project duration	file name or folder structure	EXIF/IPTC/XMP	AudubonCore compatible description in CMS
**Specimen related**	Unique Identifier	UID encoded in filename	UID encoded in filename and EXIF/IPTC/XMP	DarwinCore compatible description in CMS
	Location	file name or folder structure	DarwinCore compatible description in CMS	
	Collection data	Labels or registers imaged together or parallel with specimens	Verbatim transcription of labels	DarwinCore compatible description in CMS
	Taxonomy	file name or folder structure	DarwinCore compactible description in CMS	
	Acquisition metadata	Labels or registers imaged together or parallel with specimens	DarwinCore compactible description in CMS	
	Other specimen data	Labels or registers imaged together or parallel with specimens	DarwinCore compatible description in CMS	

Table 4. Data management requirements

**Image Data Management**	**Quality levels**		
	**1**	**2**	**3**
file names	unchanged S*/P*/R*	descriptive S**/P**/R*	naming conventions S***/P***/R**
allowable characters	no restrictions S-/P*/R-	alphanumeric, "-", and "_" S***/P***/R***	
folder structure	discretionary S*/P*/R*	manually created following conventions S**/P**/R**	automatically maintained following conventions S***/P***/R***
temporary data storage	local, no backup S-/P*/R-	local, with local backup S**/P**/R**	local, regular network backup S***/P***/R***
permanent data storage	removable media S-/P*/R*	local S-/P*/R-	dedicated archivable network area S***/P***/R***

Image quality criteria

General principles

In order to provide acceptable and repeatable image quality two main principles must be followed: consistency and documentation.

Consistency

Image acquisition conditions, post-processing protocols, metadata schemas and data management processes must be as identical as possible through the lifetime of the project, unless there is a strong reason otherwise.

Documentation

Initial protocols of image acquisition, processing, metadata collection and data handling must be documented and kept with project data. Any changes to protocols must be recorded and original documentation amended to reflect this.

Notes on technical image criteria

Composition

If possible, the whole sensor (frame) should be use to capture the object, empty space should be minimised. However, for the sake of speed it might be practical to capture images at the same magnification (zoom) even when they vary slightly in size. In the case of digitisation projects it is good practice to capture the specimen together with its collection label(s). When it is not possible, labels must be captured separately and linked to the object images using special workflows.

Focusing

It is recommended to use manual focusing; autofocus should be avoided, except when no other options are available (automatic slide scanning, for example). When the depth of focus of an instrument is inadequate, a series of images must be captured at different, slightly overlapping (~15%) focal planes and submitted for post-processing to produce an extended depth of field image (EDF, see below).

Illumination

Preferably daylight, simulating as close as possible to D50/D65 illuminant (colour temperature 5003K/horizon light or 6504K/midday light in Western Europe) should be used for photography. Full spectrum daylight fluorescent bulbs and high-CRI (colour render index) daylight LED are good choices.

Mixing different light sources must be avoided.

White balance

White balance must be setup manually except when this function is not available and accurate colour rendering is not critical. White balance and colour calibration (if applicable) must be adjusted each time the illumination changes.

ISO

Native sensor sensitivity (ISO) (Canon: 100, Nikon: 160, Fuji and most mirrorless cameras: 200) must be used unless it compromises image capture (imaging fast moving objects or at very low light). Higher or lower values of ISO are results of in-camera processing of original data

(opto-electronic conversion function) and may lead to lower signal-to-noise ration. Capture at higher sensitivity or use of different methods to increase gain (e.g. binning) may introduce digital artefacts.

Exposure

Exposure must be estimated correctly, overexposed or underexposed images are not acceptable particularly if not saved in RAW format since they lead to irretrievable information loss. Use of the histogram is the most convenient and accurate way to calculate exposure.

Scales and targets

Scales and targets (such as colour checker) should be visible on the image next to the object whenever possible. However, in cases when the size of the object prevents it, scales should be calculated through capturing separate images of a physical scale at the same magnification. Microscopes, as a rule, are calibrated, and capture software can insert virtual scales automatically. See also notes on Extended Depth of Field.

Colour space

Colour space is a particular organisation of colours that allows translation between different devices (reproducible representation of colours on different devices). Wider colour space does not necessarily mean better quality image.

sRGB

Used on most displays, cameras, scanners and printers, as well as the Internet. The sRGB color space is well specified and is designed to match typical home and office viewing conditions, rather than the darker environment typically used for commercial color matching. If the colour space of the image is unknown, sRGB is the safe choice.

Adobe RGB (1998)

The Adobe RGB (1998) color space was designed to encompass most of the colors achievable on CMYK color printers and as such is the best choice for images that will be printed on photo inkjet printers. The Adobe RGB (1998) color space encompasses roughly 50% of the visible colors, so it is slightly wider than sRGB.

ProPhoto RGB

The ProPhoto RGB color space encompasses over 90% of possible surface colors in the CIE L*a*b* color space, and 100% of likely occurring real world surface colors. It is important to keep in mind, however, that ProPhoto RGB should not be used for 8-bit per channel images.

Bit depth

JPEG files are 8-bit (24-bit, counting the three colour channels). Raw data from the sensor of a camera or a scanner can be 12- or 14-bit. To use the raw images, they have to be converted into 8-bit JPEG or TIFF images or 16-bit TIFFs (48-bit). While 16-bit images may contain more colour and dynamic range information, these are imperceptible to the human eye, as a rule. Accurate conversion algorithm will not compromise the quality of the image used for visualisation of object features. If images are used for quantitative analysis, reducing bit depth is not recommended.

File formats

JPEG files from the camera must be saved at the highest available quality. 8-bit TIFF files can be compressed using LZW or ZIP algorithms. This saves up to 60% of disk space and does not affect image quality; although may increase processing times slightly. For 16-bit TIFF files, ZIP produces much smaller files that LZW. Layered TIFF files should be avoided. The file size is very large, saving time is very long, and most printers will have trouble handling layered TIFF files. Adobe Photoshop PSD file format is efficient for layers.

Aperture

Aperture, NA, *f*-stop, etc. are related concepts referring to the size of the opening of the image-forming lens. The wider it is the more light reaches the sensor, and therefore the easier is to record the image. However, larger opening (high NA or low *f*-stop) results in a shallow depth of field and increased vignetting. High values of aperture increase a depth of field (see below) but may deteriorate resolution due to diffraction. As a good compromise it is recommended to keep *f*-stop between 5.6 and 11. For best results check the MTF (modulation transfer function) charts for a particular lens.

Depth of field

Depth of field or focus range is the distance between the closest and furthest object or detail that appears sufficiently sharp. Depth of field is roughly inversely proportional to aperture and size of the sensor (that is the larger the aperture and sensor size the shallower is the depth of field). Magnification and focal length effectively change the numerical aperture, therefore they are also inversely proportional to the depth of field. Extended depth of field (focus stacking, z-stacking, EFI) technique can be used if the necessary depth of field cannot be achieved optically (see below).

Resolution

Resolution, the ability of an instrument to capture fine detail, must not be confused with pixel count (what most people following salesmen call resolution) nor sampling rate (printing/scanning resolution). Resolution is determined by optical properties of the instrument’s image forming lens (mainly numerical aperture) and physical qualities of the sensor (size of matrix, pixel count, presence and structure of colour array filter [Bayer or other types] etc.). In order for a detail to be discernable it has to have a length of ~2.3 pixels (see Nyquist/Shannon sampling theorem for details). This means that a sensor with 6,000 pixel wide would record only ~ 3,000 black-and-white line pairs if a perfect optical system is used.

Pixel array

The pixel array is the physical number of individual sensor elements in a sensor matrix. The number of pixels has only a secondary effect on resolution: if the optical system of the instrument is not able to resolve fine details, they will not be recorded by the sensor. On the other hand, the sufficient sampling rate (at least 2 pixels per dot) must be adequate to the full advantage of optical system.

Sampling rate (scanners)

During scanning (or printing) the object, the ability of an imaging instrument to resolve fine details does not depend on the distance between the object and the instrument, and thus, can be expressed in absolute figures (ppi or dpi, pixels or dots per inch). In the case of photography and photomicrography the distance to the object is not set, so only the angular size of details tell us about the resolving power of an instrument. In these cases it is more intuitive to think of

resolution as the minimum resolved distance. It is important to distinguish between the real resolution of the instrument (data recorded by a sensor) and interpreted resolution (result of post-processing of raw data). Although absolute numbers for the latter may be higher, it may not reveal additional details compared with former.

Post-processing

Ethical guidelines for post-processing

It is recommended to follow Ethical guidelines for post-processing[3] when dealing with research images. In short, the following principles should be adhered to:

- Minimise manipulation
- Edit non-destructively
- Processing should be consistent and documented

Natural and artificial colour

Artificial colouring is generally acceptable only for images taken in greyscale (SEM, CT, laser scanning etc.). All cases of colouring must be documented.

Raw processing

Focus stacking

Focus stacking is necessary when the depth of an object exceeds the depth of field of the selected imaging method and instrument and all details of the object must be sharp. In such cases multiple (up to several hundred) images may be taken at consecutive focus planes from which a completely focused picture can be reconstructed in special software (see Appendix 1). For best results depths of field of neighbouring images should overlap slightly (~10-15%). During reconstruction images must be aligned and scaled if necessary; this can happen automatically. Helicon Focus and other software oriented on photography does not take into account perspective distortion (does not rescale image to make sure that objects in the foreground and background are the same size). Therefore EDF images from photography-oriented EDF programs (Helicon Focus, Zerine Stacker, CombineZ, Photoshop, etc.) are more pleasing aesthetically but less accurate scientifically. Physical scales, if used, must be located at the bottom focal plane of the specimen. If precise measurements are to be obtained from stacked images, EDF algorithms bundled with scientific acquisition software may be more reliable. Thorough comparison of performance of f the various focus stacking packages available has not yet been carried out.

Tiling

3D reconstruction

3D reconstruction methods may include photogrammetry, reconstruction from stereo pairs (or tilting), CT, confocal microscopy, thin section reconstruction and surface scanning (or laser scanning).

---

[3] Cromey, 'Avoiding Twisted Pixels'.

Metadata quality criteria

The operator should record metadata at the time of acquisition, or as soon as possible after acquisition, (e.g. at the end of the day). Three categories of metadata are necessary:

Project related

- Date
- Project
- Operator
- Copyright/licence information

Instrument/method related

- Instrument name/configuration
- Imaging conditions*
    - Lighting
    - Magnification
    - Special techniques

Specimen related

- Specimen UID (barcode or registration number)

---

* - if applicable

EXIF editors

File embedded metadata, such as EXIF/IPTC/XMP information can be edited within image management software, such as Adobe Bridge or Adobe Lightroom. Bulk editing of images is possible with ExifTool (command line software) and various GUI available for the program.

Audubon Core

The Audubon Core Multimedia Resource Metadata Schema (simply “Audubon Core” or “AC”) is a representation-free vocabulary for the description of biodiversity multimedia resources and collections. Its implementation in the NHM will depend on future development of KE EMu and MAMS.

Data management principles

Data management conventions are not set in stone, and can be changed from project to project if necessary. However it is vital to agree on these beforehand and maintain them through the life cycle of the project, unless change will significantly improve productivity. Below are some examples of data management agreements from current DCP projects.

Naming conventions

iCollections

Files are named IMGXXXX_NAME (where XXXX: sequenced number assigned by the camera; NAME: operator). A script renames files to BMNHE_XXXXXXX for specimen images and BMNHE_XXXXXXX_label for cut-out images of labels.

Slide project

SatScan images: BARCODE#_LocationIRN_TaxonIRN

AxioScan and AxioZoom images: BARCODE#_xxx (where xxx is three digit consequent suffix)

eMesozoic

Folder structure

iCollections
Folder structure is as follows: Project/Drawer_#/Taxon/

Slide project

eMesozoic

Research images (IAC, SBIL, etc.)
Folder structure: Laboratory/Instrument/Year/Month/ProjectOwner/Project*

---

* - if applicable

Archiving
Images of digitisation projects should be stored until successful ingest to KE EMu. Research images (including original RAW files) should be archived on UNIFIED storage. These can be stored temporarily (for the duration of the project if immediate access to files from multiple locations is absolutely necessary) upon consultation with TS.

Ingest
If file naming and folder structure conventions are maintained consistently, automatic ingest to KE EMu and MAMS is possible. Images must be moved to KE EMu staging area, and the administrator will initiate ingest. Multimedia records for each image will be created and linked to the corresponding Catalogue record, which in turn is linked to the Taxon record and Location record. In case the Catalogue record does not exist, it will be created at this point.

Images and EMu and Data Portal
Images ingested into KE EMu will be published on the Museum Data Portal. Typically they should appear within one week. If publication is not desirable, for example, active research is being conducted on the data, an embargo period of up to 12 month can be granted.

QA/QC procedures
Quality assurance should be implemented by following the above standards consistently. It is necessary to select required quality levels for each criterion at the planning stage of the project and to adhere to them at all times, unless overwhelming evidence is obtained that they should be reviewed. All deviations must be documented immediately. Regular device-level calibration is important to ensure correct functioning of imaging equipment.

Quality control for larger projects should be tier-based.

- Immediate control is performed by an operator at the end of the day or stage (preferably on other’s results, when multiple operators available).
- Weekly random check by a supervisor or independent colleague. Percentage of checked images should be agreed in project specifications.

- Delivery check, performed by export/ingest manager. This relates particularly to quality of metadata and file management procedures.
- Final check

Appendix 1. Available software

Image acquisition software

- Canon EOS Utility*±
- ControlMyCamera*± (Canon and Nikon)
- Helicon Remote*± (Canon and Nikon)
- Nikon Camera Control Pro*±
- PhaseOne Capture Pro One± (PhaseOne)
- Zerine Stacker*± (Canon and Nikon)
- Breeze Systems* (Canon and Nikon)
- Camera RC* (Nikon)
- CamRanger* (Canon and Nikon)
- digiCamControl* (Canon and Nikon)
- DSLR Assistant*
- Kuuvik Capture* (Canon)
- TetherPro* (Nikon)
- Adobe Lightroom (depending on available add-ons)
- Phocus (Hasselblad)
- PK_Tether (Pentax)
- Smart Shooter (Canon and Nikon)
- SofortBild (Nikon)
- Sony's Camera Remote Control
- Dedicated microscopy camera software*

____

* - Known to provide Live View

± - Tested

Focus stacking

- Helicon Focus
- Zerine Stacker
- CombineZ
- Photoshop
- Various microscopy solutions

Also see http://en.wikipedia.org/wiki/Focus_stacking#Software

Tiling/panorama

Creating of panoramic images or image stitching is useful when an object exceeds the field of view of an imaging instrument. Photoshop includes several panoramic algorithms. Most microscopy

acquisition/analysis software include tiling as a special optional module. A comprehensive list of software can be found in Wikipedia:

http://en.wikipedia.org/wiki/Category:Panorama_software
http://en.wikipedia.org/wiki/Comparison_of_photo_stitching_software

Image processing and analysis

Appendix 2. Sample photographic equipment lists

A. Medium-size objects and labels (field of view 30-300 mm)

Equipment	Price	Comments
**Camera**<br>Canon EOS (e.g. 600D/750D/760D)	£500-750	enthusiast range; has to be replaced after ~100,000 shots
**AC adapter**<br>Canon ACK-E8	£60	
**Lens**<br>Canon EF-S 60 mm macro lens	£350	
**Stand**<br>Kaiser Reproduction Stand RS1 / RA1 5510	£330	
**Lightbox**<br>parts: bulbs, wires, switches, plastic	£50-70	Made in-house
**Computer**	£350	
**Second monitor**	£300	Optional
**Software:**<br>**Adobe Photoshop**	£129	Optional
**Total**	**£1640-2339**	

B. Small objects (field of view 5-23 mm), high quality photographs

Equipment	Price	Comments

**Camera**<br>Canon EOS (e.g. 600D/750D/760D)	£500-750	enthusiast range; has to be replaced after ~100,000 shots
**AC adapter**<br>Canon ACK-E8	£60	
**Lens**<br>Canon EF-S 60 mm macro lens	£850	
**Stand**<br>Kaiser Reproduction Stand RS1 / RA1 5510	£330	
**Stackshot**	£350-550	
**Flash**<br>Canon MT-24 EX Macro Twin Lite	£750	
**Power source/batteries**<br>Innovatronis SpeedFire	£90	
**Computer**	£350	
**Second monitor**	£300	Optional
**Software:**<br>**Adobe Photoshop**	£129	Optional
**Helicon Focus**	£150	Can be installed on four computers as long as not used simultaneously
**Total**	**£3430-4309**	

References

Cromey, Douglas W. 'Avoiding Twisted Pixels: Ethical Guidelines for the Appropriate Use and Manipulation of Scientific Digital Images'. *Science and Engineering Ethics* 16, no. 4 (December 2010): 639–67. doi:10.1007/s11948-010-9201-y.

Hans van Dormolen. *Metamorfoze Preservation Imaging Guidelines. Image Quality, Version 1.0.* Hague: National Library of the Netherlands (KB), January 2012. http://www.metamorfoze.nl/english/digitization.

The FADGI Still Image Working Group. *Guidelines: Technical Guidelines for Digitizing Cultural Heritage Materials*. Federal Agencies Digitization Guidlines Initiatives, 24 August 2010. http://www.digitizationguidelines.gov/guidelines/digitize-technical.html.
