# Supplementary material for: iCollections methodology: workflow, results and lessons learned
Source: Biodivers Data J. 2017 Sep 25;(5):e19893. doi: 10.3897/BDJ.5.e19893 (PMC5665009; doi:10.3897/BDJ.5.e19893)
Supplement: Supplementary material 2 — NMH Georeferencing Guidelines [file bdj-05-e19893-s002.pdf]

NHM GEOREFERENCING

Updated Guidelines based on MaNIS/HerpNET/ORNIS By Malcolm Penn

Note: The most important thing to remember that assumptions should be noted.

LOCALITY TYPE	GEOREFERENCING PROCEDURE	DETERMINING EXTENT
**Named Places**<br><br>**Named Place or Urban Area:**<br><br>locality consists of a reference to a geographic feature having a spatial extent<br><br>e.g.: "Reading"	It is best to use the geographic centre (the centroid/midpoint of both the latitude and longitude extremes) for the coordinates of named places. Use the designated centre from Google maps or Google Earth to at least ensure source consistency.	Use the radius/distance from the coordinates of the named place to the furthest point within that named place.
**Named Places**<br><br>**Remote Named Place:**<br><br>locality does not have a clear boundary shown on the map<br><br>e.g.: "Dirty Socks": The extent is 0.4 mi because the nearest named feature, Hot Springs, is 0.8 mi away.	Localities that do not have a shaded boundary or a topographic symbol for buildings shown on the map, place coordinates at the dot for that locale on the map.	The extent is half the distance to the nearest named place. (Make a note of this in comment field).
**Named Places**<br><br>**Near a Named Place:**<br><br>e.g.: "Near(by) Swindon", "vicinity of Killin" "close to Killin" "above Killin" etc.	Use the geographic centre of the named place for the geographic coordinates. Remember word Near Nr, or above are appended after the place name Worcester (Nr)	The extent will be the distance from the geographic centre of the named place to the halfway point between that geographic centre and the centre of the next nearest named place. (The named place entered into the spreadsheet should include "near", or "vicinity of", or another such modifier).
**Named Places**<br><br>**Street Address:**<br>e.g. "1 Lovington lane, Lower Broadheath, Worcester"	Locate the address using GeoRef interface with Google maps	The extent is the smallest area possible that cannot be mistaken for any other address.
**Named Places**		

**Ranch/Farm, Golf course,Quarry,Mine Estates, or Parks**<br><br>e.g. "Apple tree Farm"	Treat all as named places. If you are not able to locate them with GeoRef interface, use search engines such as Google to locate them in relation to nearby geographic entities.<br><br>If farms or Golf course appear on a map, usually only the building will be shown. Take coordinates from the buildings themselves, unless you can identify the exact border of the farm.	If you can find exact boundaries, then treat the ranch or farm as you would a named place. Otherwise, the extent is half the distance between the buildings and the next nearest named place.
**Named Places**<br><br>**Junction**<br><br>e.g. "junction of Elsham Rd. and Russell Rd.", "junction of Rio Claro and Rio La Hondura"	Locate the two roads or rivers in Georef interface or a map and obtain the coordinates of the point of intersection.<br><br>Use Streetmap/Google Earth or Google Maps to help locate the road names, as roads may not be labelled on the map you are using.<br><br>Treat the Road junction as a Precise location and enter the full junction description. Treat the river junction as a Feature and enter the full description.	Measure the extent of the junction as if it were a named place. If the extent or a road junction cannot be measured on the maps available, use the following standards:<br><br>The extent is 10 m for two-lane city streets and two-lane highways.<br>The extent is 20 m for four-lane highways.<br>The extent is 30 m for large highways with medians.
**Named Places**<br><br>**Cave:**<br><br>e.g. "Las Cuevas Cave", "Chiquibul Caverns"	Georeference the entrance to the cave.	The extent is usually the surface length of the cave.
**Named Places**<br><br>**River, Mouth of River, or Head of River:**<br><br>e.g. "River Thames", "Mouth of Severn River"	River: Make a straight line from the mouth of the river to the head of the river. Calculate the centre of this line, and place the coordinates closest to the centre of the line on the river itself. Do not use the coordinates given by gazetteers, as these points usually correspond to the mouths of the river, not the geographic centres.<br><br>River Mouth: Georeference where the river	The extent is half the length of the line drawn. Make sure to only include the portion of the river that is within the specified higher geography.

	meets a larger body of water; this is usually the point of the river with the lowest elevation.<br><br>River Head: Georeference where the river starts (usually in mountains, canyons, or lakes); this should be the point of the river that has the highest elevation	The extent is half the distance across the river mouth or head (this is usually rather small).
**Named Places**<br><br>**In between two Places**<br><br>e.g. "Between Bristol and Bath, Uk."	Georeference the midpoint between the centres of both named places.	The extent is half the distance between the centres of both named places.
**Named Places**<br><br>**Names States and Parishes**<br><br>First order Admin area, State/Province<br>e.g. Florida<br><br>e.g. Settlement has the same place name as a parish/county/commune/ municipality.<br><br>e.g. If a label clearly states 2nd or 3rd Admin area (Department/ Parish/County/Commune Municipality)<br><br>e.g. If label shows a place name which can only be a parish/county	Use the geographic centre of the State/ Province for the geographic coordinates use. Use **Province**<br><br>Make assumption that the collector means the settlement, unless the label states parish/county/commune municipality. **Georeference the settlement.**<br><br>Use the geographic centre of the parish/county/commune/municipality/ county for the geographic coordinates use. **Precise Locality**.<br><br>Follow above and maps as parish/county/ commune/municipality use **Precise Locality**.<br><br>.	The extent will be the distance from the geographic centre of the State/Province to furthest point of the Province shape using the radius tool.<br><br>Use the radius/distance from the coordinates of the named place to the furthest point within that named place.<br><br>The extent will be the distance from the geographic centre of the county/parish to furthest point of the shape using the radius tool.<br><br>The extent will be the distance from the geographic centre of the county/parish to furthest point of the shape using the radius tool.

**Offsets**<br><br>**Offset Only**:<br>**locality consists of an offset from a named place without any direction specified**<br><br>e.g.: "5 km outside Brisbane"	Record the geographic coordinates of the centre of the named place, just as you would for a "normal" named place. Precise locality is 5km outside Brisbane, so not Brisbane!	Use the extent of the named place + distance.
**Offsets**<br><br>**Direction Only:**<br>**locality consists of a direction from or within a named place without any distance specified**<br><br>e.g. "N Reading", "N of Reading"	If only a direction is given, such as "N Reading" and there is no town named "North Reading", then there is no way of knowing if the collector meant "northern portion of Reading" or "North of Reading." Find the distance from the centre of the named place (Reading) to the centre of the next nearest named place to the north. Place the coordinate at one half of the distance to the centre of the next nearest named place in the direction specified.<br><br>**Remember the Direction should be appended after the place name, e.g. Reading (North or East etc.,)**	For such localities, the extent is one half of the distance between the centre of the named place in question and the centre of the next nearest named place in the specified direction.
**Offsets**<br><br>**Offset at a Heading:**<br>**locality contains a distance in a given direction**<br><br>e.g. "50 miles E of Lima"	Assume the collector measured the distance "by air." unless stated otherwise. Use the GeoRef interface to measure 50 miles in an easterly direction from the centroid of the named place.	Use the e Calculate the extent to the next nearest place name.
**Offsets**		

**Offset Along a Path, in One Direction: locality describes a route from a named place**<br><br>e.g. "7.9 mi N Beatty, on US 95"	If "by road" is specified in the locality description, Use the line tool to follow the route.<br><br>Begin at the centre of the starting point and use the measuring tool to follow the road until you have travelled the distance given. The coordinates come from this ending point.	Use the extent of the starting point.
**Offsets**<br><br>**Offset Along a River, in One Direction**<br><br>e.g. "3 miles above Worcester on River Severn on left bank"	Treat the stream as you would a road. Above refers to upstream and below refers to downstream. Left and right sides of a river are determined from the perspective of facing downstream.	Use the extent of the starting point.
**Coordinates**<br><br>**GPS (Global Positioning System)**	When georeferencing GPS coordinates, make sure to note whether the accuracy and the datum where reported. Always record coordinates in decimal degrees and make sure we distinguish the master records by using Reading (North) or Reading (North of)	The accuracy of the GPS at the time the coordinates were recorded. If none was recorded, assume 30m.
**Coordinates**<br><br>**Latitude and Longitude Coordinates: coordinates from unknown source, given in locality description**<br><br>e.g. "36 31' 21.4" N; 114 09' 50.6" W;"	Always record coordinates in decimal degrees. Enter these coordinates as the Precise locality.	Extent is 30m
