# Supplementary material for: iCollections methodology: workflow, results and lessons learned
Source: Biodivers Data J. 2017 Sep 25;(5):e19893. doi: 10.3897/BDJ.5.e19893 (PMC5665009; doi:10.3897/BDJ.5.e19893)
Supplement: Supplementary material 3 — NHM Data Embargo Policy [file bdj-05-e19893-s003.pdf]

POLICY FOR MANAGING SCIENCE DATA EMBARGOES

SUMMARY

The NHM has committed to Open Data and Open Access principles for science data, to be consistent with UK government and European Commission objectives, funding body conditions, peer institutions and the wider scientific community. This strategy would also significantly enhance our collaborative research potential and position as a world-leading resource of natural history and biodiversity data. The argument for this approach has been laid out in detail in the 'Open by Default' NHM Digital Information Issues Paper reviewed by the Board of Trustees in 2013, and Open Access principles have already been incorporated into the NHM's intellectual property policy. The DCP Digital Licensing and Citation Policy, endorsed by Science Strategy Group in January 2015, is intended to build upon that position. Once defined, data policies should be embedded within the systems and protocols for managing internal datasets and serving them up to the public domain.

Open access principles will be applicable to the vast majority of digital assets generated by the NHM's ongoing digitisation activities. **However, there are exceptions to this rule where an entirely open access and licensing model is not appropriate**, and a consistent and quantifiable approach will be needed to handle these. One such exception is designed to protect the NHM's research competitiveness, primarily by the application of appropriate embargoes to delay public release of the data.

This document describes the NHM policy and processes for applying of science data embargoes for the NHM. It should be regarded as an exception to the 'Open by Default' rule, as defined by the overarching DCP data policy framework.

KEY ITEMS

1. **Review body:** Science Strategy Group, supplemented by the Registrar and an IP specialist and with reference to specialist advisory groups as appropriate, to act as the primary authority for reviewing applications to invoke an embargo exception.

2. **Duration:** Initial applications for embargos to have the option of an initial 12 month or 36 month duration from the delivery of a research-ready dataset.

3. **Application process:** Initial embargo applications to be approved without challenge unless there are exceptional circumstances. If no application is received, it will be assumed that the data can be released under default open terms (subject to any additional exceptions).

4. **Assessment:** Applications to be based on an assessment of the 'value' that the embargo will bring to the NHM, balanced with our obligations to release data under EU, UK government and funder expectations.

5. **Renewals:** Applications to renew embargos to require increasingly compelling arguments for the 'value' that an extension would deliver.

6. **Management:** Full details of current and historic embargos to be held in a central log, maintained by the Head of Science Administration Team.

Consultation

This policy was originally drafted in consultation with the Digital Collections Programme Board, DCP Digital Policy and Standards Project Board and Science Strategy Group. Wider consultation across Science via a Town Hall meeting and direct feedback on the draft document was used to refine the final policy document.

Version History

Version	Date	Author(s)	Description
0.3	26/06/2015	Matt Woodburn, Vince Smith	Discussion/recommendation document revised to first version of the policy document, for final approval from Science Strategy Group
1.0	04/08/2015	Matt Woodburn	Removed cover sheet for first release as public policy document

CONTENTS

Summary 1
Key Items 1
Consultation 2
Version History 2
1. Background 5
1.1. Open by Default 5
1.2. Exceptions 5
2. Science data embargo applications 6
2.1. Submission 6
2.2. Review body 6
2.3. Initial embargoes 6
2.4. Embargo renewals 7
2.5. Justification for delayed release 7
2.6. Embargo start date 7
2.7. Redaction 8
2.8. Triggers for review of existing embargoes 8
2.9. Alerts 8
3. Technical implementation considerations 9
3.1. Embargo log 9
3.2. Metadata structure 9
3.3. Metadata security and curation 9
3.4. Reporting 9
Appendix A: Embargo application form template 10
Appendix B: Decision tree for science data embargoes 11
Appendix C: Summary decision tree for default and exceptions 12
Appendix D: FAQs 13
Why have embargoes? 13
To what does the embargo policy apply? 13
When and how should an embargo application be submitted? 13
How will we ensure that the process is observed? 13
Who has responsibility for making an embargo application? 13
How long do embargoes last? 13
How many renewals can be applied to an embargo? 13

When will the embargo clock start ticking? 13
How can unnecessary delays be avoided in long term digitisation projects? 14
What if there is a delay in receiving the data? 14
Can embargoes be applied to part of a dataset? 14
How will people know when the data will be released? 14
What happens if no embargo application is made? 14
How and by whom will embargos be applied to the data? 14
How can a dataset for an embargo be defined? 14
How will information about embargoed datasets be stored and displayed? 15
How will this be applied to externally funded projects? 15
What guidance will be provided on the embargo policy and process? 15
What if there are conflicting interests in embargoing a dataset? 15
How will sensitive data be protected? 15
When will the embargo policy and process be reviewed? 15

1. Background

1.1. Open by Default

The NHM has committed to Open Access and Open Data principles, predicated on EU, UK government and funder expectations and in line with good practice within the global research community. As agreed by Science Strategy Group on 15/01/2015, aligned with outputs from the Information Management Group, the default approach for NHM research and collections data will therefore be immediate release under a Creative Commons Zero open waiver (data) or Creative Commons By Attribution open licence (images), which will be expected UNLESS the data meet a valid exception to the default rule. These exceptions are outlined in the DCP Digital Licensing and Citation Policy document.

1.2. Exceptions

Exceptions to the default open policy will prompt one or more of three basic actions:

1. **No release -** Mainly sensitive collection information and confidential documents
2. **Time-delayed release (embargo) -** Mainly research datasets where immediate release would have a negative impact on the NHM's research competitiveness and funding opportunities, or due to funder conditions
3. **Release under a non-default licence and/or copyright -** Mainly for data assets with proven commercial value, or associated third party rights

A framework for reviewing all exceptions and supporting the policies is required. **Appendix C** describes the decision path of a dataset through these exceptions, and demonstrates its release under the default open policy if no exceptions are met.

Several of the exceptions require more detailed policy definitions than have so far been agreed in the DCP Digital Licensing and Citation Policy document and decision flow diagram. This document addresses that requirement for science data embargoes, and defines a framework within which they can be handled.

2. Science data embargo applications

Science data embargoes are addressed as Exception 3 ('Research Competitiveness') in the DCP Digital Licensing and Citation Policy document. This exception deals with the delayed release of data in order to protect the research interests of the NHM and internal researchers.

The various other criteria that could potentially preclude the immediate or delayed release of digital assets are handled under other exceptions, as summarised in the decision tree for default policy and exceptions in **Appendix C**. As the decision tree demonstrates, Exception 3 assumes that all other exceptions have already been considered and potentially applied.

2.1. Submission

For each new and renewed embargo, a short Embargo Application Form should be completed and submitted to the Head of Science Administration Team. The form will provide basic details of the data and briefly outline the reason for the request. **Appendix A** shows an example template and layout for an embargo application form (the form used in the live process may deviate from this over time).

For new digitisation projects, the application should be submitted before data are generated, as part of the Data Management Plan created during the project initiation process. This process will ensure that the relevant researchers are consulted and given the opportunity to request an embargo before any data are released. Alternatively, this also provides the opportunity to waive any embargo if there is no reason to hold the data back.

Digitisation projects may be based on the enrichment of existing sub records rather than the creation of new records. In this case, the application will need to be submitted before the record statuses are changed to active specimens and so included in the Data Portal export.

2.2. Review body

Embargo applications will be reviewed primarily by Science Strategy Group, and supplemented by the Registrar and an IP specialist. If the data relates to a specialist or sensitive area of the collection, such as human remains or forensics research, there may be an additional specialist advisory group that is responsible for the review. In these circumstances, Science Strategy Group is responsible for identifying and consulting with the appropriate advisory group.

The embargo review will be adopted as a standing item on the monthly agenda, with the expectation that detailed discussion will only be required in the minority of more complex cases.

2.3. Initial embargoes

Initial embargoes on new datasets will generally be expected to pass without challenge, but it is still appropriate to handle these as exceptions rather than the default, in line with the 'Open by Default' policy. Applications for new embargos will be circulated to Science Strategy Group prior to each monthly meeting to provide opportunity for any queries or objections. If none are raised, the embargo will be considered to be approved for the period requested.

Applicants will have the option to apply for an embargo duration of **either 12 or 36 months**, or alternatively opt for the default and release the data as soon as they are available. Once an

application is approved, the applicant will be considered the responsible owner of that embargo project.

2.4. Embargo renewals

Applications to extend embargoes beyond the initial time period will receive more scrutiny and discussion at Science Strategy Group. A reminder of upcoming expiry of an embargo will be generated three months before the expiry date and emailed to the owner, allowing time to for a renewal (if required) to be proposed and reviewed by Science Strategy Group.

If a satisfactory rationale is provided and accepted by Science Strategy Group, then this will trigger a one year extension from the expiry date of the current embargo. If no renewal application has been made by the date of expiry of the existing embargo, then the data will be released.

There is no limit to the number of renewals that may be applied to a dataset, but the justification for the embargo will need to be increasingly compelling for each renewal application.

2.5. Justification for delayed release

Applications for embargo requests or renewals must be supported by appropriate justification. The criteria for acceptable justification will be agreed and applied by Science Strategy Group as the review body. These are likely to reference open data commitments, scientific and funding opportunities related both to release and non-release of data, and funder conditions.

As stated above, rationales for initial embargoes should be accepted without challenge in the vast majority of cases, with further scrutiny for renewals. A decision tree will help to streamline the review of these cases, an example of which is provided in **Appendix B**. The details of the decision tree and criteria will be defined by Science Strategy Group.

2.6. Embargo start date

This must reflect a fair opportunity for the researchers to use the data, but not delay release unduly if, for example, the data management processes involved in delivering the complete dataset are complex. To reflect this, the embargo will generally start by default on the date that data collection for the project is complete. This will normally be defined as the point when the last record has been created in the Collection Management System, and will represent the delivery of a ‘research-ready’ dataset.

An exception to this rule may be long term projects which may span years in duration. The data generated by these should be grouped into consecutive tranches, and an embargo start date set at completion of data collection for each tranche. This deviation from the norm should be agreed as part of the project’s Data Management Plan, and described on the embargo application form.

The embargo release date will be calculated from the embargo start date and agreed duration. This will generally be during the project planning process, and so estimated based on the project plan. If there is significant slippage from the estimated date, then the embargo release date for that project should be reviewed (see ***2.8. Triggers for review of existing embargoes*** below).

2.7. Redaction

Partial redaction of a dataset, involving the removal of some data items from records, is a possible method of compromise over release of data. However, this has significant implications for the complexity of data management and technical implementation within KE-EMu, MAM and the Data Portal. There may be simpler cases where, for example, data might be released but the linked images embargoed, or groups of records within a dataset are held back.

Preparation of redacted or partial datasets is likely to be more practical for individual research datasets on the Data Portal than for sections of the main NHM collection dataset. However, protocols for versioning these and applying appropriate metadata would be important.

Any plans for partial redaction of records for fields must involve consultation the Data Management team to assess the technical feasibility and resource required. This should also be included in Data Management Plans and embargo applications as appropriate, depending on the current phase of the project.

2.8. Triggers for review of existing embargoes

Certain triggers may prompt the review of an existing embargo. These might include:

- Completion of the research. In particular, on publication of a paper, publisher conditions for release of the underlying dataset will need to be observed. Researchers should be responsible for informing Science Strategy Group if their research is complete or work is published.
- Project slippage. If a digitisation project is delayed in delivering a research-ready dataset, then the proposed start date of the embargo may need to be reviewed.
- Funding or research opportunities. If opportunities for funding or further research are identified that are contingent on the release of an embargoed dataset, then the embargo should be reviewed to consider the relative benefits of releasing and holding back the data.

When a situation arises to prompt a review, this should be raised with the embargo owner (if they are not the person reporting). It should also be reported to the Head of Science Administration Team to update the embargo log and escalate to Science Strategy Group if required.

2.9. Alerts

An email reminder will be sent by the Science Administration Team to the embargo owner three months before the agreed expiry date.

New records created without a valid embargo date will be published on the Data Portal as part of the next weekly release. Technical constraints and lack of consistent metadata identifying record ownership mean that alerts prior to release are not practical, and if an embargo is required the onus therefore must be on the data owner to follow the procedure.

3. Technical implementation considerations

3.1. Embargo log

A log of embargoes will be used to generate expiry reminders, store embargo application information, and link the embargo to the relevant data in KE EMu, Data Portal, MAM and other internal data resources. The log will be maintained centrally by the Science Administration Team to provide a record of all applications, embargo statuses and dates, and reasons for approval or refusal.

3.2. Metadata structure

Basic embargo metadata will be held in KE EMu catalogue and multimedia records, including the release date, reason for embargo, unique project name and embargo owner. This metadata will be exchanged with MAM and the Data Portal to support the appropriate release of the data. Embargoed catalogue and multimedia records will be filtered out from the weekly update before reaching the Data Portal, until the release date is reached.

3.3. Metadata security and curation

Appropriate metadata security settings will be needed to ensure that embargoes cannot bypass the review process, and suitable personnel and processes will need to be identified for curating embargo metadata. For the collection dataset in EMu, this responsibility is held by the Data Management team.

Embargo metadata in KE EMu will be visible to all users, but edit privileges will be restricted to the Data Management Team, and will generally be updated in bulk on a per project basis.

3.4. Reporting

Summary information on current embargoes will be published to the Data Portal and intranet, to provide internal and external transparency on which datasets are being held back. The Data Portal dashboard will be generated from metadata included in the weekly EMu table dump, while the intranet dashboard will be updated from embargo log data by the Science Administration Team.

Appendix A: Embargo application form template

**Requested by:**		**Requested start date:**	
**Date of application:**		**Submitted to:**	
**Type:**	NEW / RENEWAL	**Duration in years:**	1 year / 3 years
**Designees:**	*List all users of the data related to the application.*	**Project name:**	
**Description of data:**	*Brief description, including:*<br>● *The type of data involved*<br>● *An estimate of dataset size and record counts*<br>● *The location of the data (KE EMu/other)*<br>● *The project that will generate the data*		
**Reason for embargo or renewal:**	*Outline the justification for the embargo request, including a clear statement of the scientific or funding opportunity that would be harmed by the immediate release of the data.*<br>*If proposing a renewal of an existing embargo, describe what has been done to realise this opportunity over the previous 12 months.*		
**Previous embargo periods:**	*Provide dates for any previous embargoes relevant to the data.*		
**External sharing conditions:**	*Describe any relevant details of data sharing and ownership conditions imposed by external stakeholders, including funding bodies, Country of Origin and Landowner conditions if applicable.*		
**Object entry and/or acquisition numbers:**	*Provide all object entry and acquisition numbers that apply to the data.*		

**Approved:**	Yes / No	**Decision date:**	
**Reason for decision:**	*If the application is refused, provide reasons for the decision.*		

Forms should be completed in full and submitted to the Head of Science Administration.

Appendix B: Decision tree for science data embargoes

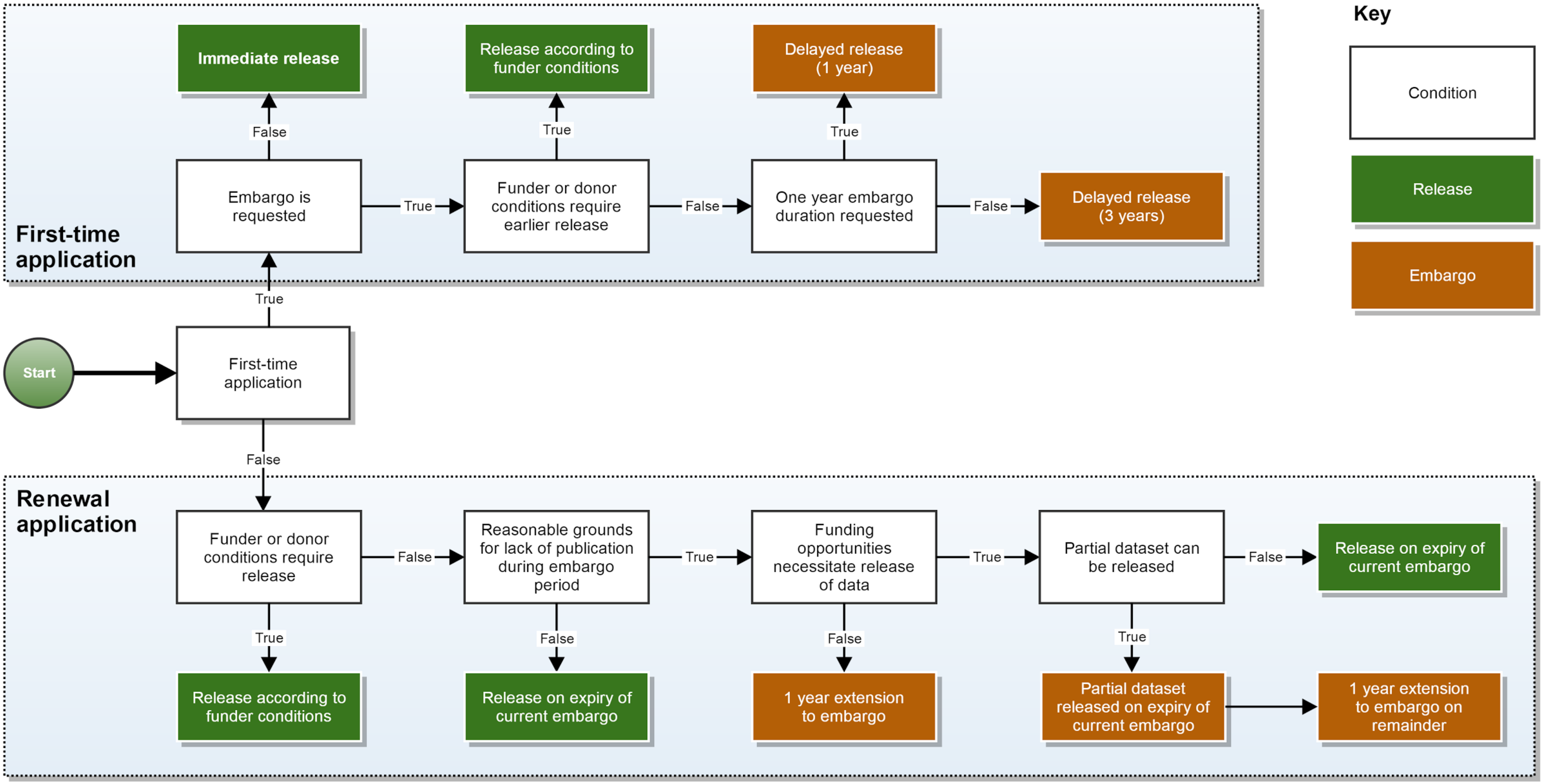

APPENDIX C: SUMMARY DECISION TREE FOR DEFAULT AND EXCEPTIONS

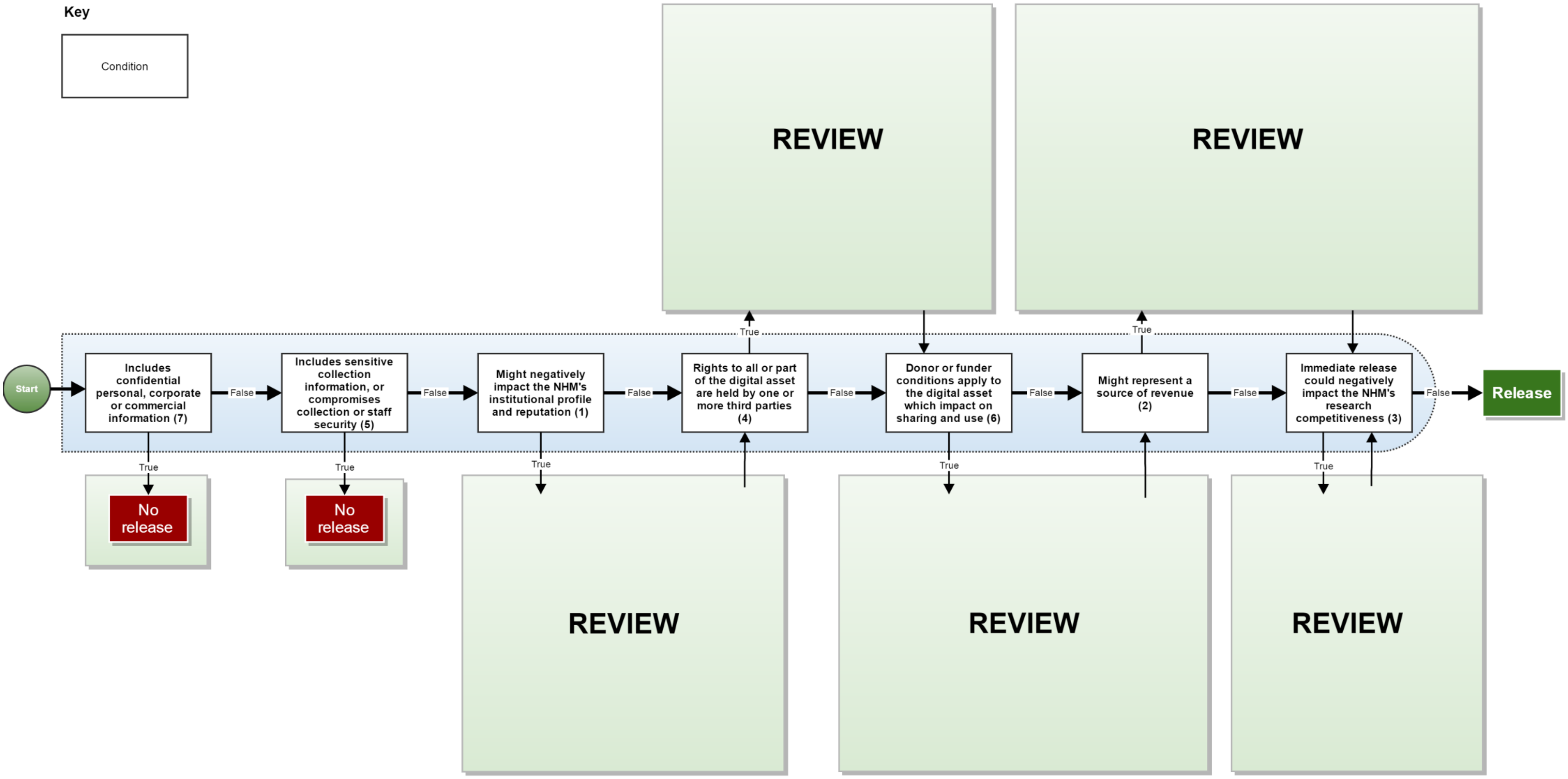

APPENDIX D: FAQS

WHY HAVE EMBARGOES?

Embargoes of limited duration are intended to provide internal researchers with a reasonable opportunity to exploit the data generated from the NHM collection for research purposes. This is an established practice within the science community, and commonly supported by UK research councils (http://www.rcuk.ac.uk/research/datapolicy/).

TO WHAT DOES THE EMBARGO POLICY APPLY?

The policy will apply to all NHM collection data.

WHEN AND HOW SHOULD AN EMBARGO APPLICATION BE SUBMITTED?

An initial application should be made during the project initiation process, on completion of a PID and Data Management Plan, and prior to the start of record creation. Applications for renewal should be submitted at least two months prior to embargo expiry date.

Applications for embargoes should be made by completing a standard form and submitting to the Head of Science Administration Team, who will summarise applications for Science Strategy Group.

Applications will need to be submitted by one week prior to the monthly Science Strategy Group meeting to allow time for circulation and review.

HOW WILL WE ENSURE THAT THE PROCESS IS OBSERVED?

Reports against the EMu database will be run periodically to check that the embargo policy and application process are being followed correctly.

WHO HAS RESPONSIBILITY FOR MAKING AN EMBARGO APPLICATION?

The research lead or SRO for each digitisation project will have responsibility for submitting the embargo application, and for discussing the details of the embargo with the relevant Data Manager. If there are external research stakeholders who have helped to compile the data, then their NHM point of contact will be responsible for any embargo application.

HOW LONG DO EMBARGOES LAST?

An application for an initial embargo may be made for either 12 or 36 months duration. Renewal applications can be made for an additional 12 months from the expiry of the existing embargo.

HOW MANY RENEWALS CAN BE APPLIED TO AN EMBARGO?

There is no fixed limit to the number of renewals. However, increasingly compelling arguments will be required to justify each renewal.

WHEN WILL THE EMBARGO CLOCK START TICKING?

By default, an embargo will apply from the date that data collection is complete. For mass digitisation projects, this date will need to be estimated before records begin to be created, and should be agreed as part of the project's Data Management Plan.

How can unnecessary delays be avoided in long term digitisation projects?

For long term digitisation projects, there may be a considerable period of time between the start of data creation and the point where data collection is complete. This could unnecessarily delay the release of data from earlier phases of the project. In this situation, the data should be split into two or more tranches, and an appropriate embargo expiry date applied to each tranche. The tranches and dates should be defined within the project's Data Management Plan.

What if there is a delay in receiving the data?

If there is an unplanned and significant delay in the delivery of a dataset, then details of the situation should be included in the application for renewal on expiry of the current embargo, should one be required.

Can embargoes be applied to part of a dataset?

It may be possible to embargo only a subset of the records involved in a dataset. Any partial embargo request should be discussed with the appropriate Data Manager to check feasibility.

Dataset redaction (i.e. removing or obscuring certain fields from the released records) would be very complicated to implement and manage in EMu, MAM and the Data Portal, and so should be avoided.

How will people know when the data will be released?

In the case of existing embargoes, the embargo log will be used to send the embargo owner a reminder three months prior to the embargo expiry date.

What happens if no embargo application is made?

If no embargo application is made, and the dataset's release is not prevented by any other exceptions to the default, then the dataset will be released as part of the next weekly Data Portal update.

How and by whom will embargos be applied to the data?

The Data Manager responsible for loading the data into EMu will add the agreed embargo end date and supporting embargo metadata to the records. Embargo metadata will be visible to EMu users on the 'NHM Sec.' tab of the Catalogue and Multimedia modules.

How can a dataset for an embargo be defined?

For mass digitisation projects, the dataset will be defined prior to creation and the records marked in EMu using project categorisation fields. In instances where records cannot be accurately grouped using their EMu metadata, a list of unique identifiers will need to be generated and stored with a link to the embargo information.

How will information about embargoed datasets be stored and displayed?

A central log of past and current embargoes and applications will be maintained by the Science Administration Team on behalf of Science Strategy Group. Summary information will be provided on the intranet to provide visibility for all stakeholders across the museum. A summary dataset will also be provided on the Data Portal for greater transparency outside of the NHM.

How will this be applied to externally funded projects?

For externally funded projects, any funder conditions or policies on data sharing and deposition will override the NHM's internal embargo policy. These will generally have a shorter maximum duration than three years.

What guidance will be provided on the embargo policy and process?

Policy documents and guides to applying for and managing embargoes will be circulated across Science, and made available to the museum in general via the intranet.

What if there are conflicting interests in embargoing a dataset?

Any conflicts of interest relating to the embargoing of a dataset that cannot be resolved between the individuals concerned should be raised with the Head of Science Administration Team, who if necessary will escalate to Science Strategy Group.

How will sensitive data be protected?

A separate exception in the policy framework has been designed to filter out sensitive collection data, prior to any consideration of research embargoes.

When will the embargo policy and process be reviewed?

These will be reviewed at the end of DCP Tranche 1, in April 2016.
