# Supplementary material for: iCollections methodology: workflow, results and lessons learned
Source: Biodivers Data J. 2017 Sep 25;(5):e19893. doi: 10.3897/BDJ.5.e19893 (PMC5665009; doi:10.3897/BDJ.5.e19893)
Supplement: Supplementary material 4 — iCollections Transcription Protocol [file bdj-05-e19893-s004.pdf]

iCollections Data Transcription Protocol

Overview of Label Data

Data on labels can be grouped into the following categories;

Collection Event Data

Collecting event data comprises the collection locality, the collection date and collector. One or more of these components may be missing. There may also be other properties of the collection locality given e.g. altitude, Ordnance Survey grid reference, a precise latitude & longitude.

Prior Collection & Sale Data

There may be one of more labels describing previous collections or sales the specimen has passed through. These data normally give the name of the prior collection or sale with no additional detail.

Registration Number and Detail

In theory all specimens in the collection should have a registration number, though in reality not all specimens will have this number. The number links to a record in the Acquisition register with details of when and from where the specimens entered the museum. This number is not unique to the specimen, rather it applies to a batch of material. The number is in the format of [museum prefix] [year]-[number] e.g. 'BMNH(E) 2013-12'. There is some variability how the prefix is written, historically it was written 'B.M.' or 'Brit. Mus.'. More recently it is written 'BMNH(E)'. On some labels the museum prefix may be missing entirely and it just gives a [year]-[number]. On the registration label it may also give the source of the registered material e.g. 'N.C. Pilleau Coll.'.

Specimen Number

A specimen number (also known as specimen barcode) is unique to a specimen and is physically associated with it. This number is to ensure any data or image can be traced unambiguously to the specimen. A small proportion of the UK Lepidoptera collection already has a specimen number allocated to them. All specimen numbers added as part of the iCollections project include a Datamatrix barcode that encodes the number in a machine readable format.

Preparation Details

These are details relating to a slide or vial preparation (or both). The information will usually be a slide or vial number but may refer to other properties of the preparation.

Taxonomic Determination

Labels from recent specimens may have information about the determination such as the name of the taxon, the person who made the determination and the determination date. However for most older specimens determination data are absent. The iCollections project *is not capturing* these data if they are present.

Type Status

A small number of specimens have type information on the labels. The values of type status encountered are likely to be; Allotype, Holotype, Lectotype, Neotype, Paralectotype, Paratype, Syntype, Type.

Additional Data

There may be present a variety of other data such as collector number, host plant data, information relating to breeding, whether the specimen was reset etc.. As these data are not part of core label data the iCollections project will not capture these.

Guidance on Data Transcription

Specimen Number

<u>Always check that the specimen number field is correct</u>

Specimen number missing or incorrect

Most records should already have the specimen number complete. If no specimen number exists then enter the number from the image. If a number is present but incorrect then:

Specimen number: enter the correct number.
Admin notes: enter 'Barcode misread' plus the incorrect number e.g. 'Barcode misread (incorrect barcode was 11199)'.

The *Specimen number* field is locked and is enabled for editing by selecting the button labelled 'e' (for edit). Once you have finished editing select the button once again to lock the field.

Occasionally when entering a specimen number an error message may display 'An error has occurred with the specimen number you are trying to edit'. This indicates a record already exists with that specimen number. This can be caused by a number of reasons that need investigating. Set *Record Status* to 'refer to admin' and *Admin comments* to 'Cannot enter missing specimen number' together with the specimen number e.g. 'Cannot enter missing specimen number 123456'.

Two specimen numbers on image

You may encounter specimens with two assigned specimen numbers (see Fig. 1). Use the lowest number as the specimen number for the record. Set *Record Status* as 'refer to admin' and *Admin Comments* as 'Image with two specimen numbers'.

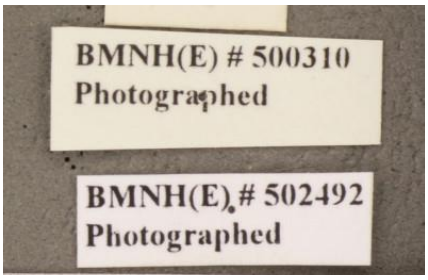

Fig. 1: Two Specimen Numbers on Image

Collecting Locality

Entering the collecting locality

- *<u>Enter the verbatim locality string</u>.* Enter the locality exactly as it appears on the label. Don't interpret or add additional information that is not on the label.
- Ignore punctuation characters such as . , ; : -
- Include Ordnance Survey (OS) grid reference, postcodes & altitude in the locality string if present.
- Separate each locality component by a semicolon.
- Enter each locality in title case (first letter capitalised).

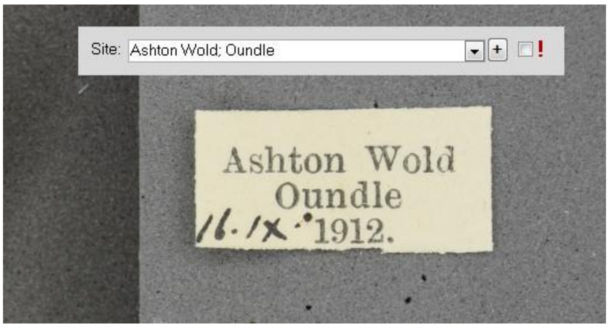

Fig. 2: Format for entering a new site

Illegible collecting localities

If all or part of the locality is illegible or difficult to interpret then tick the *locality follow-up* flag and change *Record Status* to 'needs scrutiny'. Do not use guesswork, if you are in any doubt of the locality then flag the record for scrutiny. Don't add explanatory comments into the *Label comments* field to why you can't read the label.

Location qualifiers

Location qualifiers are terms such as 'near', 'above', 'north', '1 mile from', '?' which should be captured as part of the locality data. They should be associated with the feature to which the qualification relates (see Fig. 3).

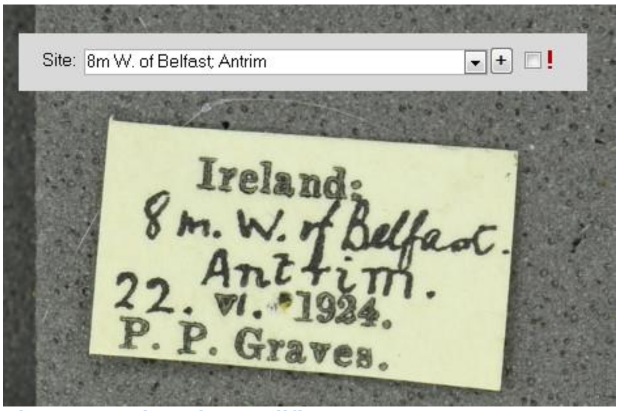

Fig. 3: Entering site qualifiers

Non British and Irish localities

Specimens from non-British or non-Irish localities (including the Channel Islands of Jersey, Guernsey, Sark, Alderney, Herm, Jethou & Brecqhou) should *not* be digitised but passed to collections staff. If you come across any of these specimens in the transcription interface then enter 'Non-British specimen' in the *Admin comments* field and change *Record Status* to 'refer to admin'.

Collection Date

Date qualifiers

The date may have a qualifier associated with it such as '?' or 'ca.' There is a separate field to record these qualifiers in front of the *Collection date from* field.

Months in roman numerals

Months written in roman numerals should be interpreted as months between 1-12.

Year consisting of only 2 digits

Where the year is written as just two digits the century needs to be interpreted as a full four digit year. In most cases the choice will be either between the 1800's or 1900's or between the 1900's and 2000's. Make a decision based on the appearance of the label and the registration date. Then tick the *Century assumed* checkbox.

Date ranges

If there is a single date then enter this into the *Collection date from* field. If there is a date range, then complete both the *Collection date from* and *Collection date to* fields.

Illegible collection date

If the collection date is illegible or difficult to interpret then tick the *date follow-up* flag and change *Record Status* to 'needs scrutiny'. Do not use guesswork, if you are in any doubt of the date then flag the record for scrutiny.

Partial dates

Entered dates should always conform to one of the following date formats;
Day/Month/Year (DD/MM/YYYY)
Month/Year (MM/YYYY)
Year (YYYY)

Sometimes you may come across a partial date e.g. the day and/or month is present but the year is missing or the day and year may be present but the month is missing. *Do not attempt to enter a partial date* e.g. if the label only states 'July' or 'Aug.191' do not enter any component of the date.

If you see a partial date and you believe the scrutiniser may be able to interpret the date then tick the *date follow-up* flag and change *Record Status* to 'needs scrutiny' and leave the *Collection Date* fields empty.

Bred & Emergence Date

There are a number of different indicators that suggest a specimen was bred (Table 1). Also as 'X' symbol with one or more dots also indicates a bred specimen.

B	B(O)	B(I)	B(L)	B(P)	bred
brood	el	em	emg	Ex	experiment
ex larva	ex ovum	ex pupa	F1	F2	F3
F4	fed	hybrid	I	Larva	ova
ovum	p	pupa	stock	X	

Table 1: indicators of bred specimen

If any of these indicators are present tick the *bred* checkbox on the *Emergence dates* tab.

There may or may not be a date on bred specimens and if present there may be one date or two dates. *If there is a single date on the label assume it is the emergence date, unless clearly stated otherwise*. Enter the date into the *Emergence date from* field. If there is a second date this will normally be the collection date and should be entered into the *Collection date from* field.

Collector

Entering collector data

Enter the data as they are stated on the label. Collector data are atomised to the following four fields; title, first, middle & last. Pick the exact match for the collector on the collector dropdown. If one does not exist then create a new collector record using the new collector form.

Multiple collectors

If there are multiple collectors then each individual collector should be entered on a separate row. Enter them in the same order they are written on the label.

Ignore full stops as part of initials

If entering a new collector record omit any full stops that are shown on the label (see Fig. 4).

Multiple middle names or initials

If there are multiple names or initials for the middle name then enter all into the middle name field (see Fig. 4).

Names consisting of only initials

Where you come across a name constructed purely of initials e.g. 'N.C.P.' then enter all the initials into the *Last Name* field. If full stops are absent on the label then add these into the *Last Name* field (see Fig. 5).

Illegible collectors

If the collector is illegible or difficult to interpret then tick the *collector follow-up* flag and change *Record Status* to 'needs scrutiny'. Do not use guesswork, if you are in any doubt of the collector then flag the record for scrutiny.

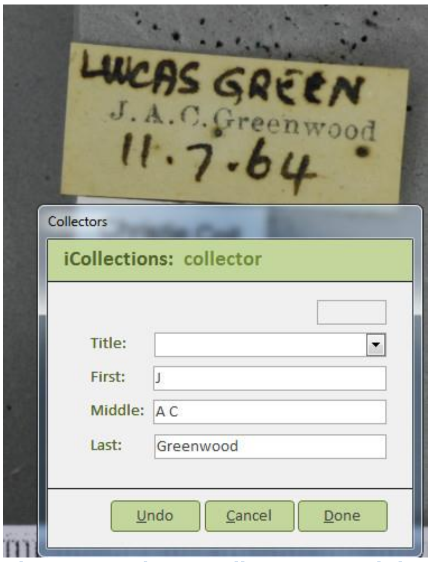

Fig. 4: Entering a collector containing initials

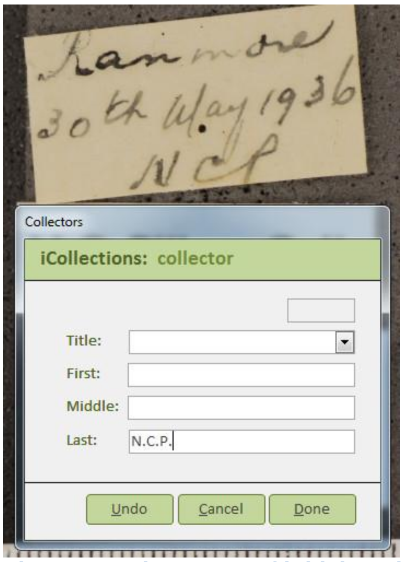

Fig. 5: Entering name of initials only

Registration Event

Enter the registration event exactly as it is written on the label. Select the name from the dropdown if it exists. If the name does not exist create a new entry by selecting the button to add a new registration event.

Registration Number

- Enter only the registration number into the *Registration no.* field. If the number has a prefix (B.M., Brit. Mus., BMNHE) leave this out e.g. the label registration ‘B.M. 1923-114’ should be entered as ‘1923-114’.
- If the year and number components are separated by a ‘.’ or ‘ ,‘ replace these characters with a ‘-‘ to ensure a consistent format (Fig. 6).

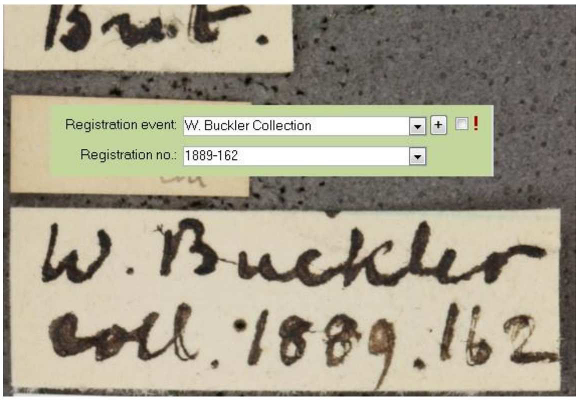

Fig. 6: Registration number format

Preparation Details

Enter the slide or vial number into the *Preparation details* field. If there are more than one preparation number then separate each with a semicolon delimiter (see Fig. 7). Rhopalocera may be abbreviated to ‘Rhop’.

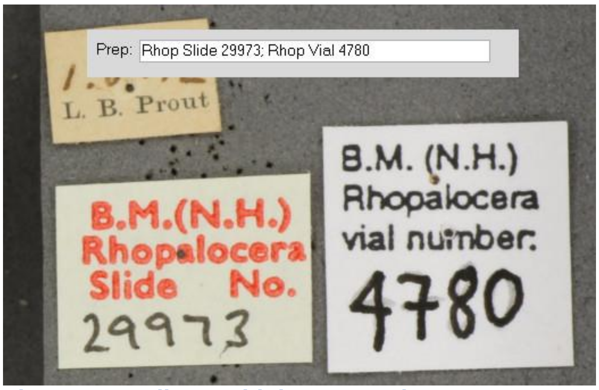

Fig. 7: Recording multiple preparations

Additional Data

If you come across any other data that is not part of the core data outlined above (this includes sex, prior collections information, sale information) *do not enter this into label comments or any other field*. However do tick the checkbox *More info on label*.

Conflict of Label Data

Conflict of collection event data

You may encounter records with a conflict of the collection event data (either collecting locality, collector or collection date). The conflict of data may be on different labels or on different sides of the same label (Fig. 8). If you come across conflicts *do not enter any of the collection event into the fields, leave them blank*. Set *Label comments* to 'Conflict of collection event data' and *Record Status* to 'complete'.

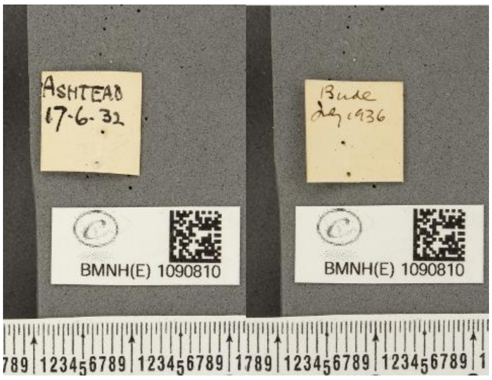

Fig. 8: Conflict of collection event data

Conflict of registration event data

Some records may a conflict of registration event data (Fig. 9). Enter the *earlier* registration event into the *Registration event* and *Registration no.* fields. In the *Label comments* field write 'Conflict of registration data' and set *Record Status* to 'complete.

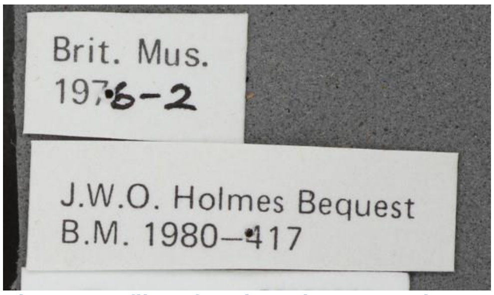

Fig. 9: Conflict of registration event data

Record Status

The *Status* field is used to indicate the record status. When you have finished editing a record change this field to the appropriate value:

none: the default value, all new ingested records are automatically set to this value. This indicates no-one has edited the record yet.

complete: once you have completed all possible fields and there are no outstanding follow-up flags checked then select this value. A shortcut to select this value is to double click the *Record Status* field, you are then taken to the next record automatically.

incomplete: if you are unsure how to interpret any part of the record and you would like to review the record at a later time then select ‘incomplete’. This is for your own personal use and allows you to easily find records you would like to review later.

needs scrutiny: if any follow up flags on the record are set and the record needs to be scrutinised by an expert e.g. the site cannot be interpreted then select ‘needs scrutiny’.

refer to admin: this value is used to indicate there is an error with the record. Refer to *Appendix A: Dealing with Errors* that gives a full list of errors and how to handle them.

Searching & Filtering Records

Finding records that are incomplete

To filter those records you have marked up as incomplete use the drop down filter ‘Status: incomplete’. To return to the full recordset simply unselect this button.

Finding a record by specimen number

Enter the specimen number in the *Find specimen number* field and press return. If the specimen number is one that is assigned to you the system will move to the appropriate record. If the number is not one assigned to you an error message will be displayed.

Finding a record by record ID

Enter the specimen number in the *Find record ID* field and press return. If the record ID is one that is assigned to you the system will move to the appropriate record. If the number is not one assigned to you an error message will be displayed.

Appendix 1: Dealing with Errors

Error	Description	Action
Barcode misread.	The specimen number has been misread and is incorrect.	*Status* to 'refer to admin'<br>*Admin comments* to 'Barcode misread' and enter the misread barcode. Then change the specimen number to the correct number
Cannot enter missing specimen number.	When attempting to enter a missing specimen number or correcting an erroneous number the error message is displayed: "An error has occurred with the specimen number you are trying to edit".	*Status* to 'refer to admin'<br>*Admin comments* to 'Cannot enter missing specimen number' plus the correct specimen number
Conflict of collection event data.	There is a conflict of some aspect of the collection event data (collection locality, collection date, collector).	*Status* to 'complete'<br>*Label comments* to 'Conflict of collection event data'<br>Do not enter any collection event data into fields!
Conflict of registration data.	There are conflicting registration events (names and/or numbers) on the label.	*Status* to 'complete'<br>*Label comments* to 'Conflict of registration data'<br>*Registration event* & *Reg. no.* fields. to the **earlier** registration
Image blurred.	The image is too blurred to read the labels.	*Status* to 'refer to admin'<br>*Admin comments* to 'Image blurred'
Image not displayed.	The image is not displayed.	*Status* to 'refer to admin'<br>*Admin comments* to 'Image not displayed'
Image upside down.	The specimen has been imaged upside down.	*Status* to 'refer to admin'<br>*Admin comments* to 'Image upside down'
Image with partial barcode.	The image has a barcode that has not been fully captured and some of the specimen number cannot be read.	*Status* to 'refer to admin'<br>*Admin comments* to 'Image with partial barcode'
Image with two specimen numbers.	There is a single image of the specimen that has two different specimen numbers.	*Status* to 'refer to admin'<br>*Admin comments* to 'Image with two specimen numbers'
Missing specimen number.	The specimen image is missing a specimen barcode label.	*Status* to 'refer to admin'<br>*Admin comments* to 'Missing specimen number'
Multiple images, all identical.	There is a single specimen record that has multiple images. The images are all identical or near identical (the same image but shifted slightly).	*Status* to 'refer to admin'<br>*Admin comments* to 'Multiple images, all identical'
Multiple images, different specimens.	There is a single specimen record that has multiple images. The images are of different specimens and have different specimen numbers.	*Status* to 'refer to admin'<br>*Admin comments* to 'Multiple images, different specimens
Multiple images, different specimens but same number.	There is a single specimen record that has multiple images. The images are of different specimens but they have the same specimen number. This happens when the same barcode has been used for two different specimens.	*Status* to 'refer to admin'<br>*Admin comments* to 'Multiple images', different specimen but same number'
Non-British specimen.	The specimen was collected from a non-British or Irish locality.	*Status* to 'refer to admin'<br>*Admin comments* to 'Non-British specimen'
Taxon name missing.	There is no taxon name.	*Status* to 'refer to admin'<br>*Admin comments* to 'Taxon name missing'
